# Development and non-clinical characterization of Procizumab (invobenitug): a humanized antibody neutralizing circulating DPP3

**DOI:** 10.1080/19420862.2026.2675077

**Published:** 2026-05-21

**Authors:** Magali Genest, Dirk Van Lier, Theo Ikenna Uba, Anika Schroeter, Adrien Picod, Hugo Nordin, Noma Assad, Gwendolyn Marguerit, Alexis Nguyen, Andreas Bergmann, Peter Pickkers, Alexandre Mebazaa, Karine Santos, Feriel Azibani

**Affiliations:** a INSERM UMR-S 942, Cardiovascular Markers in Stress Condition (MASCOT), Paris Cité University, Paris, France; b Department of Intensive Care Medicine and Radboud Center for Infectious Diseases (RCI), Radboud University Medical Center, Nijmegen, The Netherlands; c 4TEEN4 Pharmaceuticals GmbH, Hennigsdorf, Germany; d Dr. Anika Schroeter e.U, Vienna, Austria; e Department of Anesthesiology and Intensive Care, Lariboisière - Saint Louis Hospitals, APHP, Paris, France

**Keywords:** Procizumab, AK1967, Invobenitug, DPP3, circulatory failure, antibody, cardiac function, vascular function

## Abstract

Dipeptidyl peptidase 3 (DPP3) is an intracellular protein involved in cellular antioxidant responses. Upon acute stress and tissue damage, DPP3 is released into the circulation (circulating DPP3, cDPP3), where it induces vascular, cardiac, and immune dysregulation. Elevated cDPP3 levels have been implicated in multiple organ dysfunction during circulatory failure. Here, we describe the development and characterization of Procizumab (PCZ), a human immunoglobulin (Ig) G1 kappa monoclonal antibody targeting human cDPP3. The murine variant (mAb1967) was generated by immunizing BALB/c mice with a DPP3-derived peptide mimicking an exposed loop of human DPP3 and conjugated to bovine serum albumin. The humanized recombinant antibody was produced by complementarity-determining region grafting into a human framework, expressed in Chinese hamster ovary cells and purified. Binding kinetics and affinity of PCZ for human DPP3 were assessed by biolayer interferometry. The lack of Fc-mediated immune effector functions, including antibody-dependent cellular cytotoxicity, phagocytosis, and complement-dependent cytotoxicity, was demonstrated in human peripheral blood mononuclear cells. Potential cross-reactivity and specificity were evaluated using a human cell microarray assay. In vivo validation was performed in Dpp3 knock-out mice. Pharmacokinetics and tissue distribution of PCZ were assessed by quantitative whole-body autoradiography in male and female Wistar rats administered radio-labeled antibody. Finally, dose-range finding studies evaluating PCZ efficacy and safety were conducted in an acute cardiac dysfunction mouse model. Inhibition of circulating DPP3 activity represents a novel therapeutic approach for the treatment of shock, and PCZ is currently under investigation in clinical studies.

## Introduction

Shock is a clinical expression of acute circulatory failure, signifying an insufficient supply of oxygen to the cells. Shock affects one-third of intensive care unit patients.^1^ Typically, shock diagnosis is based on biochemical, hemodynamic, and clinical signs. These include systemic arterial hypotension, clinical signs of tissue hypoperfusion, and hyperlactatemia. Shock develops from several pathophysiological mechanisms, including hypovolemia (from internal or external fluid loss), cardiogenic factors (e.g., acute myocardial infarction), obstruction (e.g., pulmonary embolism), or distributive factors (e.g., from severe sepsis or anaphylaxis from the release of inflammatory mediators).^1^

DPP3 is a zinc-dependent metallopeptidase that is found mainly in the intracellular compartment in many animal species and humans.^2,3^ DPP3 is part of the central human proteome, as it belongs to a group of proteins that are ubiquitously expressed in human cells.^2^ Intracellular DPP3 activates the Keap1-Nrf2 antioxidant pathway, and it has been shown to be overexpressed under oxidative stress in a mouse model of acute cardiac dysfunction.^4,5^ DPP3 has also been detected in several extracellular compartments, including seminal plasma, retroplacental serum and cerebrospinal fluid.^6–8^ Studies suggest that the mechanism of entry of the enzyme into these extracellular compartments is either a result of its release due to injury or death of the originating cells,^9,10^ or secretion via prostasome-like membranous bodies.^8,11^ DPP3 present in the bloodstream, also known as circulating DPP3 (cDPP3), degrades extracellular physiological hormones, its most prominent substrate being angiotensin II.^2,12^ Natively purified human DPP3 administered to healthy mice results in reduced cardiac contractility, decreased vascular resistance, and impaired renal function.^4^ Additionally, intravenous DPP3 administration has been shown to significantly increase renal blood flow mediated by diminished activation of the angiotensin II type I receptor, due to reduced concentrations of circulating angiotensin II.^13^

Based on the results of these aforementioned studies, the humanized monoclonal anti-cDPP3 antibody invobenitug (Procizumab (PCZ), AK1967) was developed. Preclinical studies of PCZ in acute cardiac stress and circulatory failure models with elevated cDPP3 concentration revealed beneficial hemodynamic effects, including improvement of cardiac, vascular, and renal functions.^4,14,15^

The proposed mechanism of action of PCZ relies on binding and inhibition of cDPP3 and consequent abrogation of excess vasoactive peptide degradation (such as renin-angiotensin-aldosterone system (RAAS) peptides) and possibly other substrates involved in cardiac positive inotropism. When PCZ was administered as an intravenous bolus in mice, it promptly normalized both cardiac functions and kidney hemodynamic in an acute cardiac dysfunction mouse model. There was also significant reduction in oxidative stress and inflammatory signaling in the diseased mice.^4^ In a rat model of septic shock-induced cardiac dysfunction, PCZ inhibited cDPP3, and restored cardiac contraction and survival.^14^ In a swine model of septic shock, PCZ, administered on top of standard resuscitation, was associated with a reduced need for catecholamine support, lower lactate, decreased myocardial workload, and reduced myocardial injury. Additionally, fluid balance was reduced and oxygenation was improved compared to standard resuscitation alone.^15^

In this work, we performed a detailed characterization of PCZ’s epitope, binding kinetics, specificity *in vitro* and *in vivo*, as well as potential off-target effects. Pharmacokinetics (PK) were investigated in rats using radio-labeled PCZ. Finally, a proof-of-concept study in a mouse model of isoproterenol-induced cardiac dysfunction was also performed.

## Results

### Antibody production and characterization

PCZ’s murine variant (Ab1967) was originally generated via immunization of BALB/c mice with a DPP3 peptide conjugated to bovine serum albumin (BSA). The peptide sequence contained an additional N-terminal cysteine residue and 20 residues corresponding to an exposed loop in the sequence of human DPP3.^16^ PCZ is the humanized recombinant monoclonal variant of the murine antibody (mAb1967) generated by complementarity-determining region grafting (Syd Labs, Inc.) and produced in Chinese hamster ovary cells. The PCZ purification process included Protein A chromatography followed by a virus inactivation, and a polishing step consisting of an anion-exchange membrane with a consecutive multi-modal chromatography step. Production of PCZ yields a uniform, monomeric product of expected size following purification. The antibody undergoes ultra- and diafiltration during which it is concentrated to 20 mg/ml and stored at 2–8°C under temperature-controlled. PCZ contains a typical disulfide bridge pattern and Fc glycosylation profile at N296 site (Supplemental Figure S1).

The DPP3 epitope bound by PCZ was determined with the Seqitope® platform, a method applying massive parallel mutagenic scanning by combining phage display, affinity enrichment, next-generation sequencing (NGS) and a proprietary bioinformatic tool to determine the epitope at the single amino acid residual level. The epitope mapping results showed that PCZ specifically binds to the 7-residue sequence INPETGE. Here, the most important residues are G482, followed by P479, E480, T481, and I477, while residues N478 and E483 contribute to a lesser degree.

Binding kinetics and affinities of PCZ for human DPP3 were determined by biolayer interferometry. The representative sensorgram for PCZ is shown in Supplemental Figure S2. PCZ showed strong, sub-nanomolar affinity for DPP3 (Kd of 9.6 × 10^−9^ M), like the parental murine antibody.

**Figure 1.**
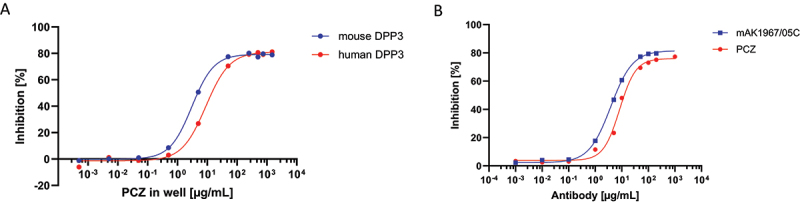
Inhibition curve of (A) mouse DPP3 vs PCZ & human DPP3 vs PCZ. (B) human DPP3 vs PCZ & human DPP3 vs mouse AB mAK1967/05C.

To confirm that PCZ inhibits DPP3 activity, an *in vitro* inhibition assay was performed with natively purified human and mouse DPP3 using a concentration range of PCZ. PCZ produced a potent inhibition of DPP3 activity in the two species, with an IC_50_ of 5.0 µg/mL for the native human target and 3.3 µg/mL for mouse DPP3 (Figure 1A). These results confirm that PCZ cross-reacts with DPP3 from mice. In addition, PCZ shows a similar inhibition to its parental murine antibody (Figure 1B).

**Figure 2.**
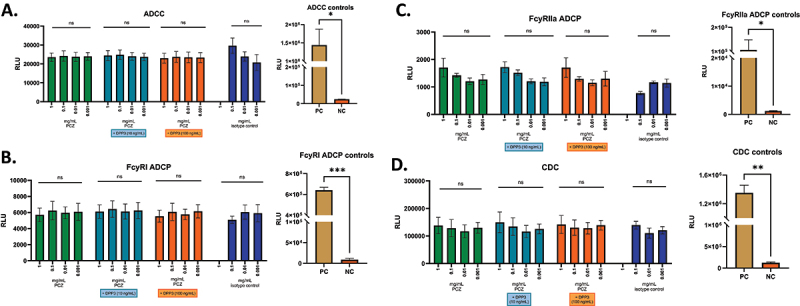
Results of the a) ADCC, B) ADCP FcyRI, C) ADCP FcyRIIa and D) CDC cell-based reporter assays. Mean luminescence signals from three donors are presented with Procizumab (PCZ) tested either alone (green; 0.001–1 mg/mL) or with DPP3 (turquoise and orange; 10 and 100 ng/mL); results from the isotype control are shown in dark blue negative control (w/o analyte; NC) and positive control (anti-CD20 IgG1 antibody; PC) are displayed in a separate graph. ADCC – antibody-dependent cell mediated cytotoxicity; ADCP – antibody-dependent cellular phagocytosis; CDC – complement-dependent cytotoxicity; FcR – Fc receptor.

To show the absence of unwanted Fc-mediated effects, the binding properties of PCZ to different Fc receptors (FcγR I, FcγR IIA / IIB, IIIA / IIIB and FcRn) and C1q was evaluated in comparison to an antibody with known specific binding to those targets (positive control: Cetuximab). The *in vitro* investigations showed a potential for PCZ binding to all investigated Fc receptors, as well as C1q (Table 1). Consequently, the potential of PCZ to induce antibody-dependent cellular cytotoxicity (ADCC), antibody-dependent cellular phagocytosis (ADCP) or complement-dependent cytotoxicity (CDC) has been investigated in the absence and presence of DPP3 levels reflecting physiological conditions in healthy humans (10 ng/mL) and supra-physiological conditions, which may occur in diseased patients.^17^ All assays were considered valid based on results of the controls. PCZ was not found to have effects related to ADCC, ADCP or CDC in human peripheral blood mononuclear cells (PBMCs) (Figure 2) confirming the absence of unwanted activation of the immune system via these pathways.

**Table 1.** Overview of results from Fc receptor screening assay and C1q binding assay.

Receptor	Results
FcγRIA	EC_50_ = 0.033 µg/mL
FcγRIIA (H allel)	EC_50_ = 0.10 µg/mL
FcγRIIA (R allel)	EC_50_ = 1.4 × 10^−27^ µg/mL
FcγR IIB	EC_50_ = 191.6 µg/mL
FcγRIIIA (V allel)	EC_50_ = 41.99 µg/mL
FcγRIIIA (F allel)	EC_50_ = 238 µg/mL
FcγRIIIB	No curve fit was possible; Binding to receptor could be shown
FcRn	EC_50_ = 0.72 µg/mL
C1q	curve fit was possible; Concentration-dependent binding could be shown

EC_50_ – Half-maximal Effective Concentration; FcR – Fc receptor.

### Specificity of PCZ

#### Whole proteome analysis and screening for off-target effects

A whole human proteome analysis was done to evaluate if the identified epitope motif of PCZ occurs in other proteins than DPP3 in the human proteome. The motif INPETGE was identified exclusively for the target, i.e., cDPP3. Subsequently and to confirm specificity of PCZ, an off-target binding analysis was conducted. Following a screening for binding against fixed HEK293 cells expressing 6105 individual full-length human plasma membrane proteins, secreted and cell surface-tethered human secreted proteins, as well as a further 400 human heterodimers, PCZ showed a specific interaction with DPP3 (Supplemental Figure S3), the primary target, and additional specific interactions with Erythrocyte Membrane Protein Band 4.1 Like 3 (EPB41L3), Killer Cell Lectin-Like Receptor G2 (KLRG2), Protocadherin 1 (PCDH1), Paraoxonase 1 (PON1) and Protocadherin Gamma Subfamily B, 2 (PCDHGB2), on fixed cell microarrays only (Table 2).

**Table 2.** Identified “specific hits” in the library and Confirmation screens: gray shaded (-) field indicate no interaction with PCZ; green shaded areas represent results with interaction from very weak (+) to strong +++; DPP3, Dipeptidyl Peptidase 3; EPB41L3, Band 4,1-like protein 3; KLRG2, killer cell lectin-like receptor subfamiliy G member 2; PCDH1, Protocadherin-1; PON1, serum paraoxonase / arylesterase 1; PDCHGB2, Protocadherin gamma-B2.

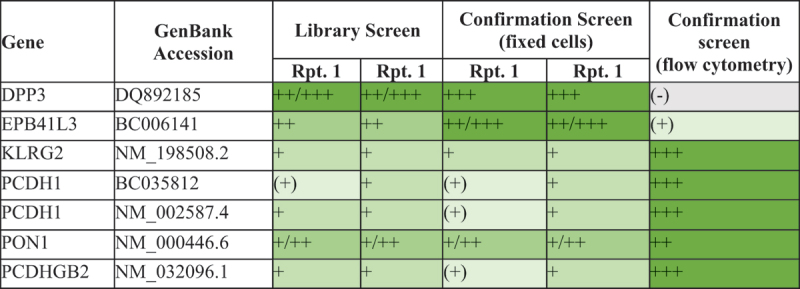

When these additional interactions were investigated further in a follow-up flow cytometry study, PCZ showed strong binding intensity with KLRG2, PCDH1 (GenBank accession numbers BC035812 and NM_002587.4), PCDHGB2 and FCGR1A (the latter Fc-domain mediated as also seen by positive control Rituximab and thus not PCZ-specific), and medium binding intensity to PON1 (Supplemental Figure S4). No significant binding was observed for DPP3, the primary (intracellular) target, and only weak to very weak binding for EPB41L3 (Supplemental Table S1).

Based on proteome analysis, PCZ’s INPETGE epitope is not present in these five potential off targets. The ‘PETGE’ motif snippet was identified in only one of the off targets, PCDH1. In addition, “PETG” is present in PON1 (Supplemental Table S2). None of the other off-targets had other epitope snippets as the “ETGE” or “ETG” motif.

#### Mutational analysis via Seqitope® and mock deep mutational scan

As a next step, we conducted a mock mutational analysis to explore potential tolerated mutations in the binding motif to elucidate binding to the observed off targets. In deep mutational scanning (DMS) experiments assessing binding preservation, tolerated substitutions are generally influenced by amino acid physicochemical properties, including side-chain chemistry, size, and charge.^18^ Conservative substitutions between residues with similar physicochemical properties are more likely to preserve binding, whereas substitutions that alter charge, polarity, or hydrophobicity often reduce binding only partially rather than abolishing it completely.^19–21^ With this information and based on the amino acid Binding Contribution (BC) score/ratio obtained from the Seqitope®, a mock DMS was performed to identify possible amino acid mutations, based on the 20 naturally occurring amino acids. (Supplemental Figure S5).

Using the BC ratios of the amino acids obtained using Seqitope®, a mock DMS for PCZ’s epitope ‘INPETGE’ was performed to identify mutations and motifs with potential binding ability (Supplemental Figure S5). After identifying these motifs, their presence in the reported off targets from the fluorescence-activated cell sorting were further investigated. Applying the AAX mutational screening results, potentially tolerated mutations of these motifs with high BC ratio were also identified and, where present, highlighted in the off-targets (Supplemental Table S2).

Applying the mock DMS, no tolerated ‘INPETGE’ mutations were identified. Possibly tolerated ‘PETGE’ mutations were identified for PON1 (as ‘PETGD’) and PCDHGB2 (as ‘PESGD’). Potentially tolerated ‘ETGE’ mutations were identified for PON1 (as ‘ETGD’) and PCDHGB2 (as ‘ESGD’ and ‘ENGE’). Furthermore, all off-targets, except PCDH1, carried putatively tolerated ‘ETG’ mutations (Supplemental Table S2).

#### Pharmacokinetics of [^125^I]-Procizumab in Wistar rats

[^125^I]-PCZ PK profile and tissue distribution was evaluated via quantitative whole-body autoradiography in rats after administration of a single intravenous (IV) dose. PCZ was radiolabeled with iodine-125 using the N-succinimidyl-3-iodobenzoate ligand method and presented satisfactory stability in rat plasma with radiopurity of about 90%. Percentage free iodide in plasma at 1 h was 0.3% for male and 0.4% for females. At 168 h, the percentage free iodide in plasma had increased to 2.1% for males and 3.5% for females (Supplemental Table S3).

#### Plasma and whole blood pharmacokinetics

**Figure 3.**
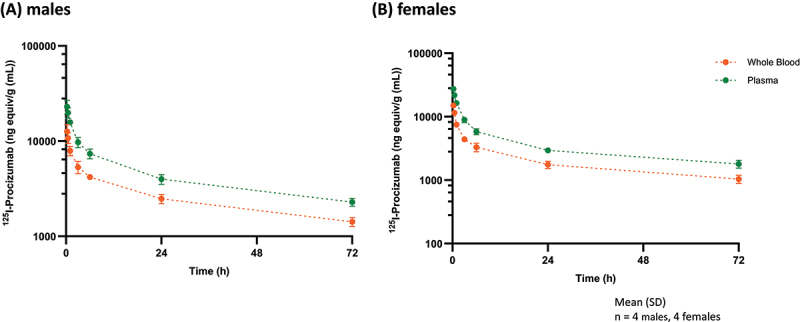
Mean concentration of total radioactivity in whole blood and plasma following a single intravenous administration of [^125^I]-Procizumab to rats. A) males; B) females.

Following a single IV administration at 1.9 mg/kg to Wistar rats, [^125^I]-PCZ exhibited biphasic distribution through 72 hours in whole blood for both males and females (Figure 3). The PK parameters calculated from mean values of total radioactivity in either whole blood or plasma, after a single IV administration of [^125^I]-PCZ, are shown in Table 3. Radioactivity derived from [^125^I]-PCZ was eliminated from sampled plasma at half-lives between 38 and 55 h (males) and 38–48 h (females).

**Table 3.** Mean (SD) values of pharmacokinetic parameters from total radioactivity in whole blood and plasma, after a single i.v. administration of [^125^I]-Procizumab. AUC_0-t_, area under the concentration versus time curve from the start of dose administration to the last observed quantifiable concentration using the linear trapezoidal method; C_max_, maximum observed concentration; n.D. – not determined; T_max_, time after dosing at which C_max_ was observed; T_1/2_, apparent terminal elimination half-life. *median value.

	Whole Blood				Plasma			
Sex	Tmax* (h)	C_max_ (ng equiv / mL)	AUC_0-t_ (ng equiv* h/mL)/	T_1/2_ (h)	Tmax* (h)	C_max_ (ng equiv / mL)	AUC_0-t_ (ng equiv* h/mL)	T_1/2_ (h)
Male	0.25(-)	12,600 (2250)	192,000 (11400)	42–53 h (n.d.)	0.25 (n.d.)	23,000 (3850)	324,000 (5470)	38–55 h (n.d.)
Female	0.25(-)	15,000 (476)	148,000 (15400)	41–45 h (n.d.)	0.25 (n.d.)	27,200 (973)	265,000 (17800)	38–48 h (n.d.)

Systemic exposure to total radioactivity (based on mean C_max_ and AUC_(0-t)_ estimates) was similar between males and females in plasma and whole blood (Supplemental Table S2). Female/male ratios of mean C_max_ and AUC_(0-t)_ were 1.2 and 0.8 for both matrices. A trend for systemic exposure to total radioactivity in plasma to be greater than in whole blood was noted in males and females, with whole blood/plasma ratios of C_max_ and AUC_0-t_ ranging from 0.5 to 0.6 (Supplemental Table S4).

#### Tissue distribution

**Figure 4.**
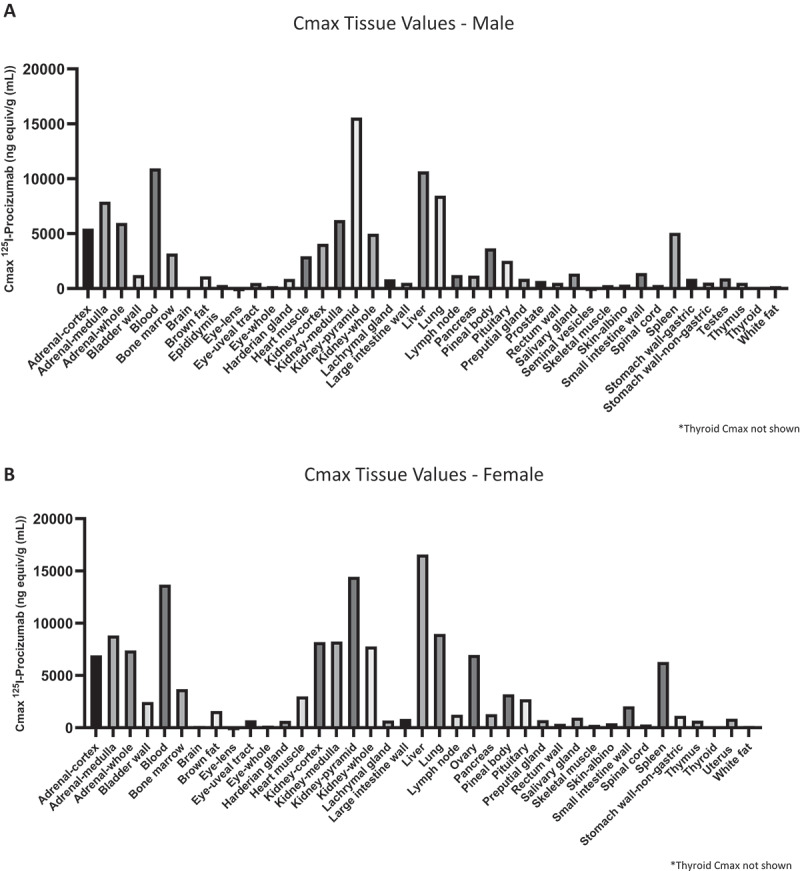
Cmax concentration (ng equiv/g) for all investigated tissues (except for thyroid) in the male (A) and female (B) animal after single i.v. injection of [^125^I]-Procizumab.

Distribution of total radioactivity was widespread and measurable in all tissues (Figure 4A, B). The concentration of total radioactivity was highest in the blood (10,939 ng equiv/g for males and 13,691 ng equiv/g for females) and liver (10,674 ng equiv/g for males and 16,578 ng equiv/g in females). Concentrations were also relatively high (i.e., >5000 ng equiv/g) in the lungs, adrenal medulla and cortex, kidney pyramid, and spleen in both sexes. In addition, ovary levels were measured at a level of 6953 ng equiv/g. All other tissue concentrations, except for thyroid, were in the range of 25–<5000 ng equiv/g in males and 56–<5000 ng equiv/g in females. Levels in the thyroid increased over time reaching 79,052 and 47,883 ng equiv/g in males and females, respectively. Tissues with comparably low signal (<200 ng equiv/g) even at - <5000 ng equiv/g in females. Levels in the thyroid increased over time, reaching 79,052 and 47,883 ng equiv/g in males and females, respectively. Tissues with comparably low signal (<200 ng equiv/g) even at C_max_ were eye and brain in both sexes and seminal vesicles for males.

In most tissues sampled, the maximum observed concentration (C_max_) was attained at the 0.25 h or 1 h timepoint, except most notably the thyroid with a T_max_ (time at which C_max_ was observed) at 24 h in males (Supplemental Table S5A) and at 72 h in females (Supplemental Table S5B). Generally, concentration of total radioactivity rapidly decreased during the first 24 h post dose and slowed down thereafter. At 168 h (last sampling timepoint), signals remained detectable in all tissues. This tissue retention could be attributable to excretion being incomplete at 168 h (Supplemental Table S6). The main route of radioactivity excretion was via the urine with 61% in males and 66% in females recovered at 168 h. Feces excretion accounted for only ≤4%.

### Procizumab effect on cardiac function in vivo in Dpp3-knock out (DPP3^−/−^) mice

**Figure 5.**
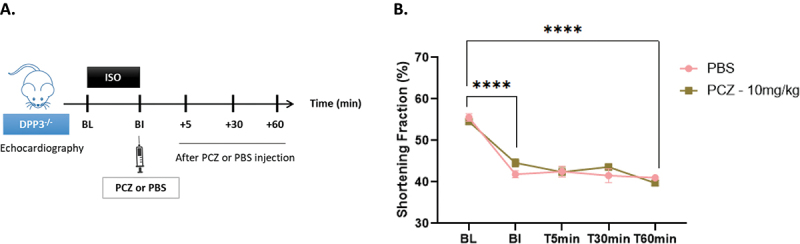
Cardiac function of DPP3 knock out mice injected with Procizumab (PCZ). A: protocol design scheme; B: fractional shortening (FS) over time of mice receiving an i.V. bolus of PBS-1X or Procizumab (10 mg/kg). Abbreviations: bl, baseline; BI, before injection.

PCZ specificity was further validated *in vivo* in isoproterenol-induced cardiac dysfunction model using DPP3^−/−^ mice. Fractional shortening (FS) of DPP3^−/−^ mice receiving either PCZ or PBS was comparable over the time (Figure 5A,B) suggesting that PCZ-induced improvement of cardiac function in wild-type (WT) mice ^4^ resulted from direct inhibition of DPP3 activity.

### Dose-range finding efficacy studies

**Figure 6.**
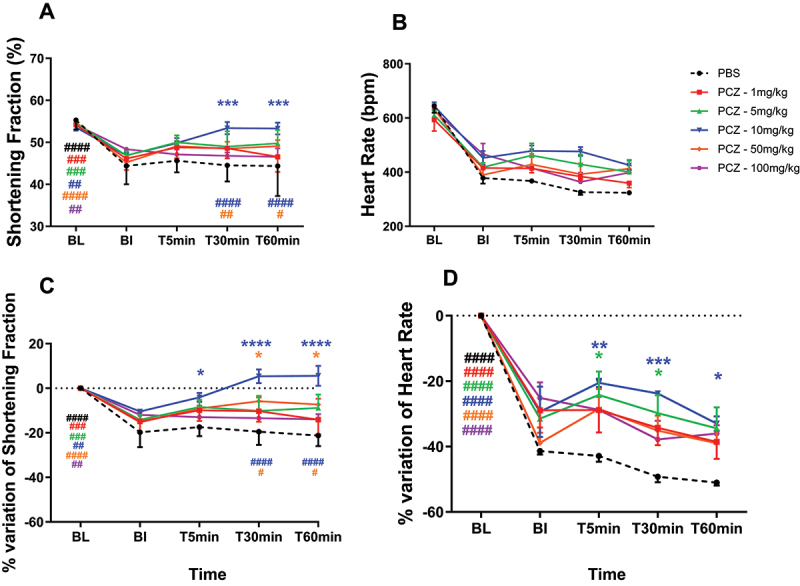
Mice cardiac function in efficacy protocol. Fractional shortening (A), heart rate (B), percentage of variation of shortening fraction (C) and percentage of variation of heart rate (D) of isoproterenol- induced acute cardiac dysfunction in mice injected with PBS or Procizumab (PCZ). *: <0.05; **: <0.005 vs PBS. #: <0.05; ##: <0.005; ###: <0.0005; ####: <0.0001 vs BI.

The isoproterenol-treated WT animals included in the different groups had at Baseline (BL) a mean decrease in shortening fraction (SF) of 14.09% compared to before treatment or placebo injection (BI). Phosphate-buffered saline (PBS) injection did not modify the SF of isoproterenol-induced cardiac dysfunction mice (Figure 6A,C). However, PCZ administration restored normal cardiac function in diseased mice in two of the doses tested (10 and 50 mg/kg) (Figure 6A,C). The injection of PCZ at 1, 5, and 100 mg/kg did not modify the SF of acute cardiac dysfunction mice (Figure 6A,C). Concerning the heart rate, isoproterenol-injected mice were more sensitive to anesthesia and presented with decreased heart rate levels (Figure 6B,D) that increased post PCZ treatment, but less pronounced post PBS treatment. All dose levels were well tolerated based on body weight and clinical signs assessment (data not shown).

## Discussion

PCZ is a first-in-class humanized monoclonal PCZ that neutralizes cDPP3 with high affinity and specificity. Epitope mapping identified a short linear motif (INPETGE) uniquely present in the human proteome within DPP3, providing strong molecular justification for target selectivity and supporting further clinical development.

A comprehensive off-target assessment identified five potential binders (KLRG2, PCDH1, PCDHGB2, PON1, and EPB41L3) in a microarray-based screen. Only two of these proteins (PCDH1 and PON1) contained partial epitope motif snippets, and mock DMS suggested that tolerated mutations of motif fragments (e.g., PETGE or ETG) may account for the weak binding signals observed in HEK cell-based assays. Importantly, bioinformatic analyses and literature review indicated that these proteins are well conserved across mammals, implying that biologically relevant off-target effects would likely have been detected in preclinical species, including DPP3^−/−^ mice. With the exception of EPB41L3, which is predominantly intracellular,^22^ the remaining candidates are theoretically accessible to circulating antibodies.^23–29^ Nevertheless, no *in vivo* evidence of off-target toxicity was observed.

As expected for an IgG1 antibody, PCZ retained the capacity to interact with Fc receptors and complement component C1q.^30^ However, functional assays showed no induction of ADCC, ADCP, or CDC, indicating the absence of undesired Fc-mediated effector functions. These findings are further supported by repeat-dose toxicology studies in mice and cynomolgus monkeys^31^ and a Phase 1 clinical study in healthy volunteers,^32^ which collectively demonstrated favorable tolerability without signals of off-target binding or immune activation.

Consistent with typical monoclonal antibody PK, PCZ showed predominant distribution within the vascular compartment, where cDPP3 is present and pharmacological activity is expected. No sex-specific or tissue-specific accumulation was observed, arguing against clinically relevant off-target engagement. High radioactivity in well-perfused organs following [125I]-PCZ administration was consistent with blood pool distribution, while thyroid uptake reflected physiological iodine handling.^33^ Urinary radioactivity resulted from PCZ degradation products or free iodine, in line with established monoclonal clearance mechanisms.^34–36^

Pharmacodynamic studies demonstrated that PCZ doses ≥ 5 mg/kg effectively inhibited cDPP3 activity and that 10–50 mg/kg significantly reversed cardiac dysfunction in a murine model of isoproterenol-induced injury. These findings align with the emerging pathophysiological role of cDPP3 in circulatory failure, a condition affecting nearly one-third of intensive care patients and still associated with high short-term mortality despite contemporary management.^1,37–39^

Elevated cDPP3 concentrations have consistently been reported across multiple shock etiologies, including ischemic and non-ischemic cardiogenic shock, septic shock, hemorrhagic shock, and major burns, where they correlate with disease severity and mortality.^4,40–45^ Importantly, cDPP3 kinetics appears to be a robust prognostic marker independent of shock etiology, underscoring the clinical relevance of targeting circulating enzymatic activity.

Despite its short plasma half-life, sustained elevation of cDPP3 likely reflects ongoing tissue injury and oxidative stress.^3,4^ Strong correlations between cDPP3 concentration and enzymatic activity indicate that the protein circulates in an active form and that endogenous inhibition is negligible in critically ill patients.^3,14^ In large reference cohorts, cDPP3 concentrations are low, with median values around 15 ng/mL and an upper normal limit of approximately 30 ng/mL. When expressed in molar terms and compared with PCZ exposures at pharmacologically active doses in preclinical models (5–10 mg/kg) and in the Phase 1 study (3–12 mg/kg), circulating antibody concentrations exceed cDPP3 by several orders of magnitude and remain above the in vitro IC50 for cDPP3 inhibition for prolonged periods. Together with GLP compliant toxicology studies showing no adverse findings suggestive of off target engagement at supratherapeutic PCZ exposures, these data indicate that antigen concentrations are unlikely to saturate available binding sites in vivo and that PCZ exposure, rather than antigen limitation, is the main driver of target engagement and safety margin in the intended clinical setting

Among known substrates, angiotensin II (Ang II) is probably the most physiologically relevant and plays a central role in cardiovascular homeostasis.^46^ In shock, compensatory Ang II production is increased, yet its bioavailability may be compromised by impaired angiotensin-converting enzyme activity, endothelial dysfunction, and enhanced cDPP3-mediated degradation.^47–50^ Persistently elevated cDPP3 activity may therefore exacerbate vasoplegia, myocardial depression, and renal dysfunction through excessive Ang II cleavage.^4,13^

Taken together, these data support a mode of action whereby PCZ mitigates circulatory failure by inhibiting cDPP3, thereby preserving Ang II bioavailability during critical illness. This hypothesis is reinforced by preclinical studies demonstrating improved RAAS balance, hemodynamics, and organ function following PCZ treatment.^13,15^

The targeted DPP3 epitope is highly conserved across species, enabling translational evaluation in both small and large animal models. Beyond confirming previously reported efficacy in murine cardiac dysfunction,^4^ the study presented here defines a therapeutic window combining efficacy and safety, which informed dose selection for subsequent GLP toxicology studies,^31^ large animal experiments,^15^ and first-in-human evaluation (NCT06331884).^32^ Our work has some limitations. First, although our off-target profiling encompassed over 6,500 human plasma membrane, secreted, and cell surface-tethered proteins, it does not exhaustively cover all intracellular proteins that may be released into the circulation under pathological conditions. Given that DPP3 gains access to the extracellular compartment upon cellular injury, the possibility that other damage-associated intracellular proteins could interact with PCZ in plasma cannot be formally excluded. Future studies using complementary plasma proteome-wide interaction approaches would be valuable to more comprehensively characterize the off-target landscape of PCZ under pathological conditions. Second, the mock deep mutational scanning analysis presented here is an exploratory, in silico approximation that lacks explicit structural and epistatic context and should therefore be interpreted only as a qualitative, hypothesis generating indication of potential epitope similarity rather than as a definitive predictor of residue level binding tolerance. Last, given the high sequence conservation of DPP3 between species and the demonstrated reduction of circulating DPP3 activity by PCZ in rat plasma, we assumed comparable target engagement in the rat PK and biodistribution studies.

While the current work is limited to cell‑based assays and murine models, and we focused our assessment on selected cardiac endpoints in the preclinical dose‑range finding study, the collective data nevertheless position PCZ as a promising therapeutic candidate, and ongoing clinical evaluation in cardiogenic shock patients (PROCARD 1b/2a, NCT06832722) will provide a more comprehensive assessment of its dose-response profile and clinical potential.

## Materials and methods

### Antibody generation

Generation, screening, and development of the humanized DPP3 antibody PCZ have been described previously.^4^ The antibody used in all experiments was provided by 4TEEN4 Pharmaceuticals GmbH (Hennigsdorf, Germany).

### Antibody characterization

#### Disulfide linkage and free cysteine analysis

Disulfide bond mapping was performed on two protein batches under non-reducing conditions to identify bridged cysteine residues. Samples were first denatured, followed by alkylation of free cysteines using iodoacetamide to prevent artificial disulfide formation. Subsequently, proteins were digested with a combination of endoproteinase Lys-C and trypsin. To simplify analysis, N-glycan moieties were enzymatically removed prior to mass spectrometric measurement. The resulting peptide mixtures were analyzed by reversed-phase ultra-performance liquid chromatography coupled with ultraviolet detection and electrospray ionization quadrupole time-of-flight tandem mass spectrometry (RP-UPLC-UV-ESI-QTOF-MS/MS) using collision-induced dissociation. Peptide identification was performed through in silico generation of peptide lists based on theoretical tryptic cleavage sites. Data interpretation focused on the detection and verification of disulfide-linked peptides through comparison of measured precursor ion masses (MS) and corresponding fragmentation spectra (MS/MS).

#### Epitope mapping via Seqitope

To determine the epitope of PCZ, epitope mapping was conducted using Seqitope, a proprietary high-resolution, high-throughput method developed by AAX Biotech, Sweden.^51^ As a first step, a large library of the target antigen DPP3 containing point mutations was generated by combining error prone PCR and display on phage. After production of the mutated antigen displaying phages, the library was mixed with PCZ immobilized onto a solid support. Unbound phages and their displayed antigens are removed by washing, while phages with tolerated mutations in the binding regions are retained. The samples were then subjected to NSG to obtain information regarding which specific phage captured PCZ (i.e., which mutation was capable of binding to the antibody).

Data analysis was performed using Seqitoper^TM^, a custom-built analysis pipeline developed in-house at AAX Biotech, which determines the contribution of binding for each amino acid residue presented as a BC ratio/score. A high score indicates importance to binding. The theoretical BC value for noncontributing amino acid residues is 0. For each position and each amino acid, a ratio is calculated between the read count in the PCZ-panning condition and the corresponding read count in the reference. Binding ratios above 0.5 are classified as potentially tolerated for antibody binding at that position, while ratios below 0.5 are deemed unfavorable for potential binding at said position.

#### Inhibition assay

DPP3 inhibition by PCZ was measured using a soluble activity assay (SAA) as described below (*DPP3 activity measurements* section). Here test samples were prepared with a known amount of either native human DPP3 or mouse DPP3 and mixed with a dilution series of PCZ. Test samples were pipetted into uncoated, black 96-well microplates. To start the reaction, assay buffer (50 mM Tris/HCl, pH 7.8 (25°C) with 0.125% Triton X-100) was mixed with an artificial substrate for DPP3 (Arg-Arg-β-naphthylamine) and added to the wells. The plate was incubated for 60 minutes at 37°C in the dark. The fluorescence of the cleaved product βNA (*t* = 60 min) was detected using the Twinkle LB 970 fluorometer (Berthold Technologies GmbH) using an excitation and emission filters of 340/10 nm and 420/10 nm, respectively. Finally, the signal expressed as relative fluorescence units (RFU) was plotted against the corresponding antibody concentrations. The data points were fit using a 4-parameter logistic regression model to determine the IC_50_ using GraphPad Prism software.

#### Binding kinetics

To determine the affinity of PCZ to DPP3, the association and dissociation kinetics of DPP3 binding to immobilized antibody were determined by means of label-free surface plasmon resonance using a Biacore 2000 system (GE Healthcare Europe GmbH, Potsdam, Germany). PCZ was assessed for binding against biotinylated human DPP3. PCZ was loaded onto Anti-Human Fc (AHC) biosensors. Loaded biosensors were dipped into an antigen dilution series (300 nM, 1:3 down, 7 points) to observe association. The sensors were then dipped into assay buffer to observe dissociation. Kinetic analysis was performed using 1:1 binding model and global fitting. Antibody binding kinetics were determined by standard procedures, and affinity constants were calculated (Biaffin GmbH, Kassel, Germany).

#### Screening for Fc-receptor/ C1q interactions

The binding properties of PCZ to different Fc receptors (FcγR I [Sino Biological; Cat# 10,256-H08H], FcγR IIA [Sino Biological; Cat# 10,374-H08H] / IIB [Sino Biological; 10,374-H08H1], IIIA [Sino Biological; Cat# 10,389-H08]/ IIIB [Sino Biological; Cat# 11,046-H08H]and FcRn [Sino Biological; Cat# CT009H08H]) and C1q ((Merck Milipore; Cat# 204,879-1 MG) was evaluated in comparison to an antibody with known specific binding to those targets (positive control: Cetuximab [BMS; Cat# 12,399,042]). For generating binding curves, a chemiluminescent-based AlphaScreen assay was used for FcγR and FcRn receptors. Briefly, this assay measures the emission at 520–620 nm in the absence and presence of PCZ. Absence of any emission at that wavelength indicates binding to the Fc receptor. Interaction with C1q was tested based on an enzyme-linked immunosorbent assay (ELISA).

#### Analysis of ADCC, ADCP, and CDC activity

The potential of PCZ to induce ADCC, ADCP, or CDC has been investigated. As a first step, human PBMCs (CRL; blood draw was done with informed consent) were selected as a relevant cell line for PCZ binding potential and intended intravenous (IV) route of administration. PBMCs from three donors were included in each assay. Three different cell-based reporter bioassays were used to investigate ADCC, ADCP or CDC potential in the absence or presence of human DPP3 (10 or 100 ng/mL). Established positive (i.e., anti-CD20 IgG1 antibody) and negative (isotype control) controls as well as a control cell line (Raji or Ramos cells) were included in each assay.

For measurement of ADCC and ADCP, commercially available ADCC, FcyRI, and FcyRIIa-H Reporter Bioassay Core Kits were used (Promega; Cat# G7018, GA1345, and G9995 respectively). Positive control anti-CD20-hlgG1 (Invivogen; Cat# hcd20-mab1) and isotype control anti-betaGal-hlgG1 (Invivogen; Cat# bgal-mab1) as well as PCZ alone or as mix with DPP3 were added at 25 µL in serial dilutions (1–0.001 mg/mL) to 96-well plates containing either 12,500 human PBMCs (assay plate) or Raji cells (control plate) per well. Effector cells included in the ADCC or ADCP Reporter Bioassay Core Kits were prepared according to the manufacturer’s instruction and 25 µL added to the assay and control plate (effector to target cell ration = 6:1). After 16–24 h of incubation at 37°C the BioGloTM Luciferase assay reagent was added according to the manufacturer’s instruction and the luminescence intensity (relative light units; RLU) determined by a Tecan Multiplex Reader and the reader Software Magellan.

The CDC assay was developed in house. PBMCs (assay plate) and Ramos cells (control plate) were pre-cultured according to standard procedures. Eight dilutions of PCZ (with or without DPP3) and isotype control (anti-bGal-hlgG1) as well as positive control (anti-CD20-hlgG1) were prepared the 30 µL transferred in the respective assay plate (1–0.001 mg/mL). Target cell suspension was added (20,400 cells/well) and the mixture incubated for 15 min at room temperature. Normal human complement serum (Teco medical; Cat# NHS; Lot#46b; 9 µL) was added to each well to induce CDC. After 4 hours of incubation at 37°C, CytoTox-Glo^TM^ cytotoxicity assay (Promega; Cat# G9291) was used to measure dead cell proteases using the Tecan Multiplex Reader and the reader Software Magellan.

### Specificity evaluation

#### Proteome analysis and mock deep mutational scan

To map the ‘INPETGE’ motif to the off-target protein (KLRG2, PCDH1, PCDHGB2, PON1, and EPB41L3), the Uniprot number of each off target was first retrieved from Ensembl.org ^52^ using the gene/protein name. Each off target full protein sequence was then downloaded from the Uniprot entry.^53^ Finally, the ‘INPETGE’ motif was scanned against the selected off target protein using the ScanProsite tool,^54^ confirming its presence (or absence) and position within the off target protein.

In the proteome analysis, the human reference proteome (Uniprot No: UP000005640, taxon 9606) was downloaded in FASTA format from Uniprot and searched for the ‘INPETGE’ motif using the *Biostrings* package in R version 4.4.2 (http://www.r-project.org). Exact matches of the motif were identified across all protein sequences with *vmatchPattern*, and proteins containing at least one match were extracted based on the number of hits per sequence.

Applying the Seqitope® tool for mock DMS, the analysis pipeline generated raw count files that report the number of DNA sequencing reads recorded for each position in the analyzed protein or peptide. These reads were categorized into conserved reads, which represent the expected amino acid at each position in the wild-type sequence, and non-conserved reads, which correspond to alternative amino acids introduced through mutagenesis. The non-conserved reads were further broken down to show, for each position, the number of reads associated with each possible amino acid substitution. This involved comparing the read counts obtained from antibody panning against a mutant library to those from appropriate reference library. For each position and each possible amino acid, a ratio is calculated between the read count in the antibody-panning condition and the corresponding read count in the reference. A ratio greater than one indicates that the given amino acid substitution is likely favorable for antibody binding at that position, with higher ratios suggesting stronger positive contributions. Conversely, a ratio below one suggests that the substitution is unfavorable for binding, with lower ratios reflecting a more detrimental impact on epitope recognition.

#### Screening for off-target effects

The Retrogenix Cell Microarray Technology Platform which enables broad screening of potential interactions with clinically relevant extracellular and circulating protein was used to screen for specific off-target binding of PCZ. As a first step, a library screening was conducted at a concentration of 0.1 μg/mL PCZ, 1 mg/mL Rituximab biosimilar (positive control; CRL High Peak, UK), or PBS (negative control) for binding against fixed human HEK293 cells, individually expressing both, ZsGreen 1 and 6105 full-length human plasma membrane proteins, secreted and cell surface-tethered human secreted proteins plus further 400 human heterodimers. The proteins were individually arranged in duplicate across microarray slides. An expression vector (pIRES-hEGFR-IRES-ZsGreen1) was spotted in quadruplicates on every slide and was used to ensure that a minimal threshold of transfection efficiency has been achieved on every slide. Detection of binding was performed by using a fluorescent secondary antibody (AF647 anti-hlgG Fc; CRL High Peak, UK) and fluorescence imaging. Fluorescent images were analyzed and quantitated using ImageQuant software (GE healthcare; Version 8.2). Two replicate slide-sets were conducted. A protein interaction was defined as a duplicate showing a raised signal compared to background levels and interactions were classified as “strong, medium, weak or very weak” depending on the intensity of the duplicate spot. Each library interaction was re-expressed, along with two control receptors (CD20 – positive control; EGFR – transfection and negative control; CRL High Peak, UK), and re-tested with 0.1 µg/mL PCZ or control treatments (1 µg/mL Rituximab biosimilar, 0.1 µg/mL isotype control) in a confirmation screen. This was performed in fixed cells (*n* = 2).

The off-target binding of PCZ to specific proteins, i.e., EPB41L3, KLRG2, PCDH1, PON1, and PCDHGB2 on live cells was examined by flow cytometry method. Expression vectors encoding the proteins were transfected, in duplicate, into human HEK293 cells. Live transfectants were then incubated with 0.1 µg/mL PCZ, and 1 µg/mL Rituximab biosimilar (assay positive control) or assay buffer only (negative control). Cells were washed and incubated with AF647 anti-human IgG Fc detection antibody (CRL High Peak, UK). The cells were again washed and analyzed by flow cytometry using an Accuri (BD). A 7AAD live/dead dye was used to exclude dead cells in the analysis, and ZsGreen+ (transfected) cells were selected for analysis.

### In vivo evaluation

#### Animals

Animal experiments were performed at INSERM UMRS-942, according to the National Institutes of Health Guide for Care and Use of Laboratory Animals and were approved by the Lariboisière-Villemin Animal Ethics Committee (2016113016181432). Healthy C57BL/6JRj male mice (9–16 weeks) provided by Janvier Labs were used. Knock-out mice for *Dpp3* gene (DPP3^−/−^) were obtained from Graz University.^55^

In addition, male and female Wistar (albino) rats (8–9 weeks old and body weight 208–306 g at the time of dosing) were supplied by Charles River, UK. The animals were acclimatized to the experimental unit for at least 7 days prior to use on the study. A standard laboratory diet of known formulation (SDS Rat and Mouse Maintenance Diet No.1, Special Diet Services, 1 Stepfield, Witham, Essex) and domestic mains tap water were available *ad libitum* for the duration of both the pre-trial and on-study periods. Holding and study areas had automatic control of light cycles and temperature. Light hours were 07:00–19:00. Ranges of temperature and humidity measured during the study were 20–22°C and 36–61%, respectively.

### Tissue distribution and plasma / whole blood pharmacokinetics in Wistar rats

#### Radiochemistry

[^125^I]-PCZ (specific activity 0.146 mCi/mg (5.38 MBq/mg), radioactive concentration 5.16 MBq/mL and radiochemical purity 99.12%) was supplied as preformulated solution in PBS by Chelatec and stored at of 2–8°C. To determine the extent of bound radioactivity, an aliquot of plasma (*ca* 0.1 g) was removed from each plasma sample at 1 h and 168 h into a centrifuge tube and to this 10 vol/g ice cold 10% trichloroacetic acid was added. The sample was vortex-mixed and placed on ice for approximately 10 min. The sample was then centrifuged (10 min at 3000 r.p.m. in a centrifuge set to maintain a temperature of 4°C) and the resulting supernatant decanted. Duplicate aliquots (*ca* 0.01 g) of each supernatant were removed for total radioactivity analysis.

#### Plasma and whole blood pharmacokinetics of [^125^I]-Procizumab

Four male and four female Wistar (albino) rats each received a single IV administration of [^125^I]-PCZ via the tail vein as a slow bolus over *ca* 30 seconds at a target dose volume of 2 mL/kg to achieve a nominal dose level of 1.9 mg/kg (target radioactive dose of 10 MBq/kg). They were group housed by sex, in polypropylene and stainless-steel cages with wire mesh floors. Blood samples (*ca*. 0.3 mL) were collected into lithium heparinized tubes by venepuncture of jugular vein at 0.25, 0.5, 1, 3, 6, 24, and 72 h post dose. PK parameters were analyzed on a Phoenix WinNonlin 6.4 software (CERTARA, Princeton, NJ) using non-compartmental methods consistent with the IV bolus route of administration. The mean (SD) values are reported for each PK parameter, except for Tmax, where the median is reported. No mean for T1/2 was calculated for samples where the extrapolation of the AUC to infinity represented more than 20% of the total area.

#### Tissue distribution and excretion

Eight male and 8 female albino rats received an IV slow bolus [^125^I]-PCZ dose of 1.9 mg/kg (target radioactive dose of 10 MBq/kg). Two male and 2 female rats, were housed singly in all-glass metabolism cages to examine total excretion radioactivity over time (until 168 h). One male and one female rat and timepoint were killed by overdose of barbiturate into the right tail vein at 0.25, 1, 24, 72, and 168 h post dose. Right before sacrifice, a sample of whole blood (*ca* 1 mL) was collected *via* the right tail vein into tubes containing lithium heparin. Duplicate aliquots (*ca* 0.025 g) of whole blood were removed from each sample for analysis of total radioactivity and plasma was prepared from the remaining sample by centrifugation (10 min at 3000 r.p.m. in a centrifuge set to maintain a temperature of 4°C) and the blood cells discarded. Duplicate aliquots (*ca* 0.01 mL) were removed from each sample for total radioactivity analysis. Immediately following sacrifice, the right eye from each animal was removed and weighed into a tared gamma counting tube for analysis of total radioactivity.

#### Evaluation of PCZ effect in DPP3-knock out mouse model

We induced cardiac dysfunction with isoproterenol as previously described.^14^ Briefly, five male DPP3^−/−^ mice were injected subcutaneously twice daily for 2 days with 300 mg/kg/day of isoproterenol (#I5627-5 G, Sigma-Aldrich). Twelve hours post-injection, mice with a cardiac dysfunction (10% decrease of SF or more) were randomized into two groups, one receiving PCZ (10 mg/kg) and the other PBS 1X in a one bolus IV injection. Cardiac function was monitored by echocardiography up to 1-hour post-injection on anesthetized animals.

#### Efficacy assessment in isoproterenol-induced acute cardiac dysfunction in WT mice

A week before the experiment, we performed baseline (BL) echocardiography under ketamine anesthesia (100 mg/kg) and a retro-orbital blood draw of approximately 200–300 µL (on dry capillary, in a heparin-lithium tube and an EDTA tube). Then, WT animals received isoproterenol as described above. Twelve hours after the last isoproterenol injection, animals were anesthetized by intraperitoneal (IP) ketamine injection (100 mg/kg) and an echocardiography was performed for 5–10 min (Before Injection or BI). Mice presenting with cardiac dysfunction (decrease of 10% in fractional shortening – FS) received PCZ or PBS bolus injection in a volume of 100 µL via the retro-orbital vein. Mice were divided into 6 groups: PBS (3 animals), PCZ 1 mg/kg (4 animals), PCZ 5 mg/kg (4 animals), PCZ 10 mg/kg (5 animals), PCZ 50 mg/kg (5 animals) and PCZ 100 mg/kg (4 animals). Cardiac function was monitored by echocardiography for a period of 15 minutes at the time-points T15min (15 min post injection), T45min, T75min. The animals were reinjected with ketamine (40 mg/kg) 5 min before each timepoint if needed. At the end of cardiac monitoring, mice were sacrificed.

#### Echocardiography

Cardiac function was measured non-invasively by transthoracic cardiac ultrasound using Vivid 7 system (General Electrics, USA) equipped with a 14-MHz linear transducer. Mice were kept on a heating mat at 37°C. Image capture was done for a period of 15 minutes. The following parameters were analyzed: telediastolic and telesystolic left ventricular diameters, the thickness of the septal and posterior walls, the shortening fraction and the heart rate.

#### Sacrifice

During sacrifice, a blood draw of around 1 mL for mice, was taken from the retro-orbital vein with a dry capillary in a heparin-lithium tube and an EDTA tube, followed by an IP injection of pentobarbital (500 mg/kg). The blood was centrifuged at 2000 g, for 10 min at 4°C for the heparin-lithium tube and for 15 min at 6°C for the EDTA tube. The plasma was then aliquoted and kept at −80°C.

### DPP3 activity measurements

The method is a one-step soluble activity assay that is run without solid capture phase and therefore requires no washing step. Analyte and reagents were incubated in black, non-binding 96-well microplates (Greiner Bio-One #655900). A calibrator dilution series was based on a dilution series of human, native DPP3 in zero matrix (heat-inactivated horse serum with 0.09% NaN_3_) and two Quality Controls were based on human, native DPP3 in heat-inactivated human EDTA plasma with 0.09% NaN_3_. 15 µL of mouse serum samples were pre-diluted in 15 µL Dilution Buffer based on 50 mM Tris/HCl, pH 7.8 (25°C) with 0.5% BSA.

Just before starting the assay, a substrate solution was prepared based on 50 mM Tris/HCl, pH 7.8 (25°C) with 0.125% Triton X-100 and the following additives with indicated target concentration taken from frozen stock solutions: 0.125 mM of a DPP3 substrate Arg-Arg-βNA (Arginine-Arginine-beta-Naphthylamide), 1 mM Cobalt chloride and 0.25 mM Amastatin. 10 µL of 1:2 diluted samples, undiluted controls and calibrator were pipetted into designated wells (duplicate determination). Then 90 µL of the substrate solution was added per well. Baseline fluorescence at *t* = 0 min was measured in a plate fluorometer with excitation wavelength of 340/10 nm and emission wavelength at 420/10 nm. The plate was immediately transferred to an incubator to support the enzyme reaction at 37°C in the dark. After 1 hour signal intensity of the fluorescent cleavage product βNA (beta-Naphthylamine) was measured again. Obtained RFU data was analyzed using Excel®. Baseline RFU values of *t* = 0 min was subtracted from the *t* = 60 min values. The mean of duplicate wells was calculated and further analyzed. Per DPP3 calibrator level signals were plotted against its respective known activity in Units per Liter (U/L) to perform linear regression forced through zero to allow calculation of U/L from RFU signals of controls and samples. Controls had to be recovered within the allowed target range to validate a run. As functional control, a βNA based dilution series was included in the runs.

### Statistical analyses

Data are expressed as mean ± SEM. In the shortening fraction study, results are further analyzed by a two-way ANOVA followed by a Dunnett’s multiple comparisons test was performed, where appropriate, in comparison to BI timepoint and to PBS group. Statistical analyses were performed using GraphPad Prism version 7.0 (GraphPad Software Inc., San Diego, CA, USA). A *p*-value < 0.05 was considered statistically significant.

## Supplementary Material

Supplemental Material

## Data Availability

Access to data may be granted upon reasonable request to qualified researchers. Data can be requested by submitting a research proposal and statistical analysis plan to the corresponding authors.
