# Supplementary material for: Development and non-clinical characterization of Procizumab (invobenitug): a humanized antibody neutralizing circulating DPP3
Source: MAbs. 2026 May 21;18(1):2675077. doi: 10.1080/19420862.2026.2675077 (PMC13196627; doi:10.1080/19420862.2026.2675077)
Supplement: Supplemental Material [file KMAB_A_2675077_SM8812.docx]

**Supplementary Materials**

**Suppl. Figure S1.** Structure of Procizumab including glycosylation sites (yellow stars) and location of disulfide bridges (S-S).

Figure S1 Alt text: Diagram of a monomeric IgG antibody composed of two heavy chains and two light chains. The structure shows the Y-shaped configuration with two Fab arms (antigen-binding fragments) at the top and the Fc region (stem) at the bottom, joined by a hinge disulphide S-S bridges. There are two stars in the CH2 region on both arms indicating the glycosylation sites.

**Suppl. Figure S2.** Kinetic profiles for Procizumab binding recombinant human DPP3 at 37°C.

Figure S2 Alt text: Figure shows kinetic data, i.e., association and dissociation curves, of PCZ binding human DPP3. The stacked curves of four concentrations for PCZ show an exponential increase in intensity for the association phase and a decrease (and almost flat at lower concentration) in for the dissociation phase. The fitting is represented in red dashed lines.

**Suppl. Figure S3.** Cell Microarray screening of concentrations of Procizumab to its primary target of DPP3 on fixed cells

Figure S3 Alt text: Picture of a cell microarray screening where the position of the bands shows the degree of binding for different concentrations of Procizumab to DPP3, and PBS.

**Suppl. Figure S4.** Flow cytometry profiles investigating specific interactions of Procizumab with selected proteins.

Figure S4 Alt text: Figure made of five graphs showing flow cytometry profiles of concentrations of Procizumab with selected proteins, and assay buffer with secondary, as well as Rituximab.

**Suppl. Figure S5.**  Results of Mock Deep Mutation Scan for PCZ’s epitope “INPETGE”

Figure S5 Alt text: Figure showing a heat map of binding contribution ratio of the “INPETGE” motif to each of the twenty native amino acids. This mock DMS highlights the contribution of binding for each amino acid residue presented as a BC ratio. A high ratio indicates importance to binding, and the theoretical BC value for non-contributing amino acid residues is 0. A BC ratio is calculated between the read count in the “INPETGE” motif and the corresponding read count in the reference for each position and each amino acid.


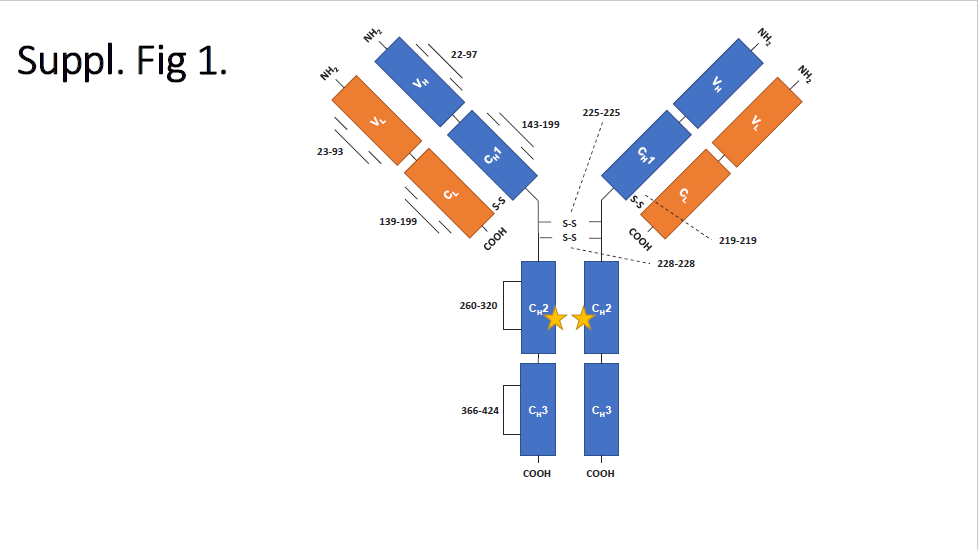


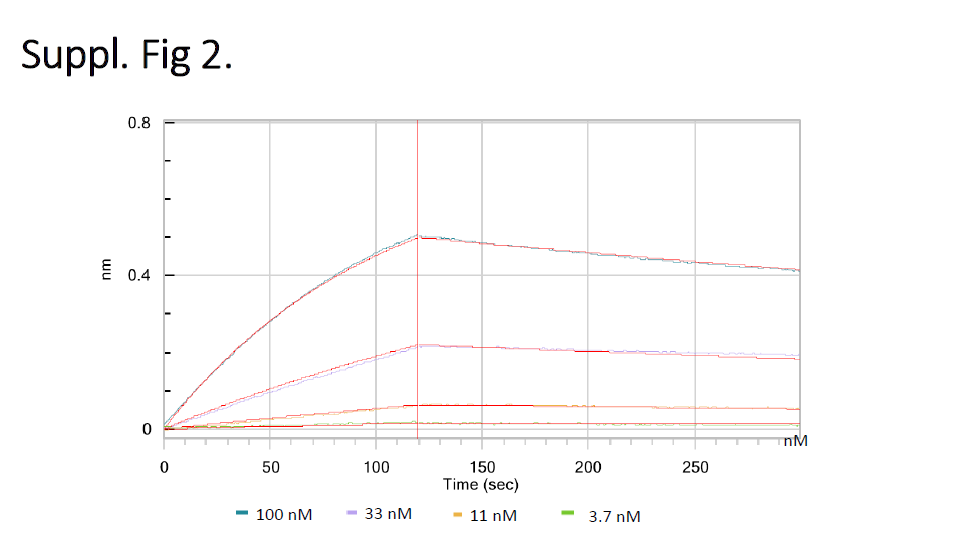


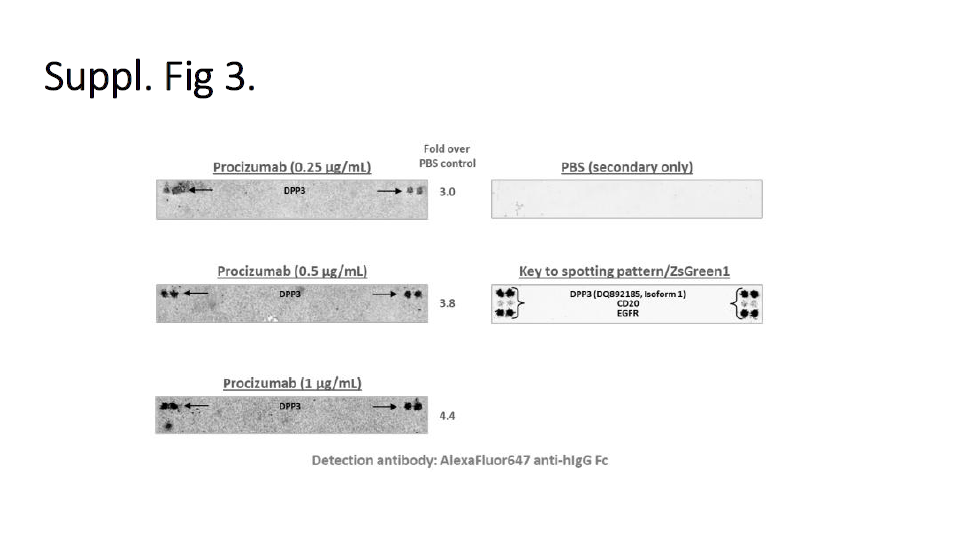


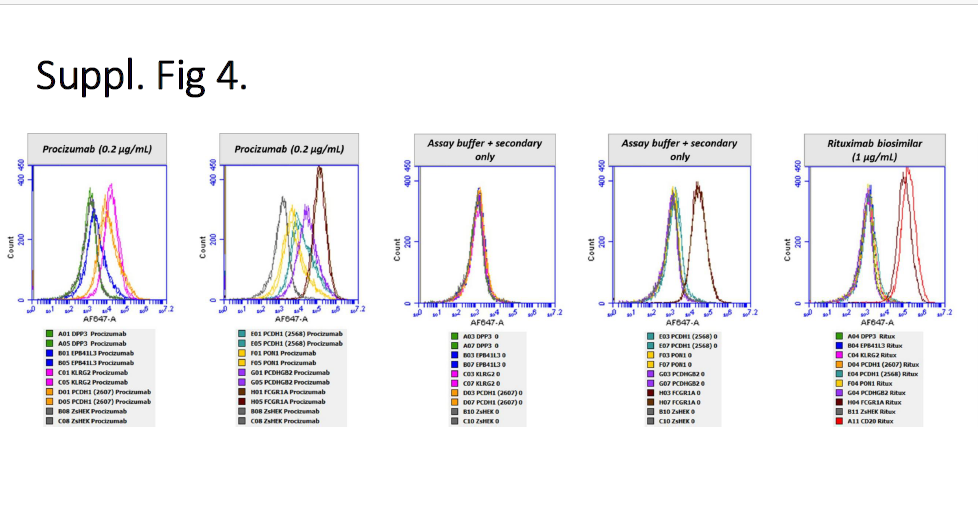


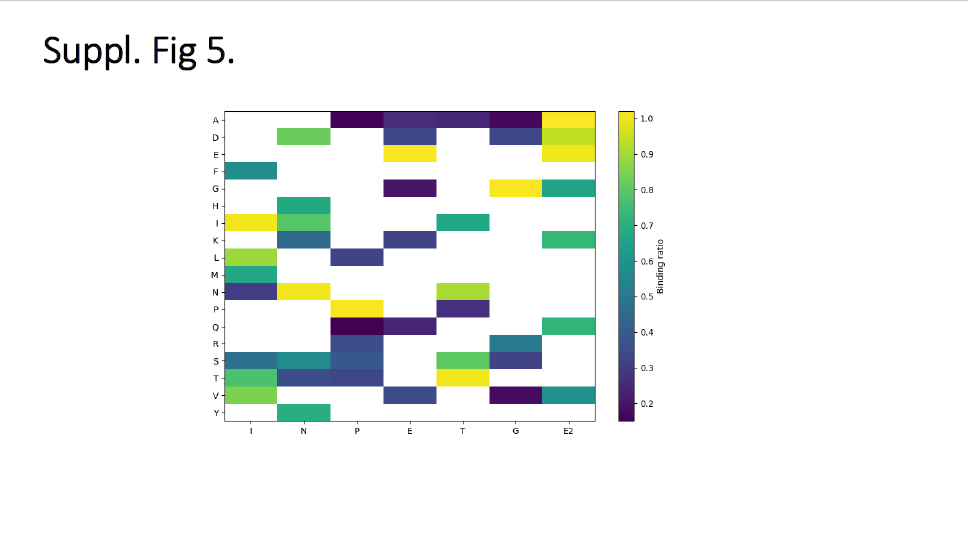


**Suppl. Table S1:** Quantitative flow cytometry data with median AF647 fluorescence (fold change from Zs-Green-only transfected HEK293s): green shaded fields indicate weak (bright green) to strong (dark green) fluorescent intensity binding; NT – Not tested.

| **Protein** | **Replicate** | **PCZ** | **Buffer** | **Rituximab** |
| --- | --- | --- | --- | --- |
| **DPP3** | R 1 | 1012 (1.1) | 982 (1.1) | 976 (1.1) |
|  | R 2 | 1069 (1.2) | 921 (1.1) | NT |
| **EPB41L3** | R 1 | 1798 (2.0) | 967 (1.1) | 1024 (1.2) |
|  | R 2 | 2335 (2.6) | 929 (1.1) | NT |
| **KLRG2** | R 1 | 34762 (38.1) | 991 (1.2) | 984 (1.1) |
|  | R 2 | 44858 (49.2) | 990 (1.2) | NT |
| **PCDH1^1^  (isoform 1)** | R 1 | 7105 (7.8) | 939 (1.1) | 941 (1.1) |
|  | R 2 | 6374 (7.0) | 869 (1.0) | NT |
| **PCDH1^2^**  **(isoform 2)** | R 1 | 3770 (4.1) | 927 (1.1) | 953 (1.1) |
|  | R 2 | 3156 (3.5) | 914 (1.1) | NT |
| **PON1** | R 1 | 2688 (2.9) | 917 (1.1) | 910 (1.1) |
|  | R 2 | 3904 (4.3) | 910 (1.1) | NT |
| **FCGR1A** | R 1 | 173608 (190.5) | 79504 (92.9) | 528064 (613.3) |
|  | R 2 | 199534 (219.0) | 86253 (100.8) | NT |
| **PCDHGB2** | R 1 | 9393 (10.3) | 782 (0.9) | 820 (1.0) |
|  | R 2 | 14192 (15.6) | 828 (1.0) | NT |

^1^Accession number: #BC035812; ^2^Accession number #NM_002587.4

**Suppl. Table S2:** Presence of the “INPETGE” motif or snippets and potentially tolerated mutations in the five identified potential off-targets.

| **Off-targets** | **Presence of Epitope or Snippets** | | | | | **Potentially Tolerated Mutations** | | | | |
| --- | --- | --- | --- | --- | --- | --- | --- | --- | --- | --- |
|  | **INPETGE** | **PETGE** | **PETG** | **ETGE** | **ETG** | **INPETGE** | **PETGE** | **PETG** | **ETGE** | **ETG** |
| **EPB41L3** | No | No | No | No | No | No | No | No | No | ESG |
| **KLRG2** | No | No | No | No | No | No | No | No | No | ESG |
| **PCDH1** | No | Yes | Yes | Yes | Yes | No | No | No | No | No |
| **PON1** | No | No | Yes | No | Yes | No | PETGD | No | ETGD | ENG |
| **PCDHGB2** | No | No | No | No | No | No | PESGD | PESG | ESGD  ENGE | ESG  ENG |

**Suppl. Table S3:** Percentage free iodide in selected plasma samples measured by direct gamma counting.

| **Animal ID** | **Time (h)** | **% Free Iodide** |
| --- | --- | --- |
| **011M** | 1 | 0.3 |
| **016M** | 168 | 2.1 |
| **019F** | 1 | 0.4 |
| **024F** | 168 | 3.5 |

**Suppl. Table S4:** Female / male and whole blood / plasma ratios for C_max_ and AUC_0-t_.

|  | **C_max_** | **AUC_0-t_** |
| --- | --- | --- |
| **Female / Male Ratio Whole Blood** | 1.19 | 0.77 |
| **Female / Male Ratio Plasma** | 1.18 | 0.82 |
| **Whole Blood / Plasma Female** | 0.55 | 0.56 |
| **Whole Blood / Plasma Males** | 0.55 | 0.59 |

**Suppl. Table S5A:** Concentration (ng equiv/g) of total radioactivity in selected tissues at 0.25, 1, 24, 72 and 168 h in males**;** C_max_ for each tissue is highlighted in yellow.

| **Tissue** | **0.25 h** | **1 h** | **24 h** | **72 h** | **168 h** |
| --- | --- | --- | --- | --- | --- |
| Adrenal-cortex | 5463 | 3825 | 880 | 388 | 206 |
| Adrenal-medulla | 7910 | 4641 | 1146 | 422 | 260 |
| Adrenal-whole | 5978 | 3993 | 900 | 388 | 217 |
| Bladder wall | 442 | 1047 | 1231 | 561 | 234 |
| Blood | 10939 | 7816 | 1679 | 928 | 627 |
| Bone marrow | 3190 | 1980 | 540 | 181 | 139 |
| Brain | 166 | 90 | 43 | 19 | 14 |
| Brown fat | 1102 | 718 | 413 | 351 | 182 |
| Epididymis | 327 | 788 | 317 | 164 | 118 |
| Eye-lens | 25 | 53 | 66 | 14 | 20 |
| Eye-uveal tract | 408 | 508 | 429 | 222 | 80 |
| Eye-whole | 154 | 182 | 222 | 92 | 76 |
| Harderian gland | 674 | 874 | 333 | 178 | 138 |
| Heart muscle | 2938 | 2381 | 708 | 377 | 236 |
| Kidney-cortex | 3060 | 4083 | 634 | 264 | 249 |
| Kidney-medulla | 3190 | 6235 | 852 | 491 | 331 |
| Kidney-pyramid | 5831 | 15583 | 1089 | 526 | 369 |
| Kidney-whole | 3431 | 4991 | 694 | 324 | 286 |
| Lachrymal gland | 620 | 839 | 441 | 149 | 150 |
| Large intestine wall | 490 | 530 | 371 | 192 | 154 |
| Liver | 10674 | 8509 | 1276 | 572 | 326 |
| Lung | 8456 | 6000 | 1279 | 659 | 541 |
| Lymph node | 1145 | 1227 | 421 | 326 | 166 |
| Pancreas | 752 | 1176 | 316 | 146 | 118 |
| Pineal body | 3659 | 1187 | 606 | 302 | 242 |
| Pituitary | 2533 | 1247 | 584 | 244 | 185 |
| Preputial gland | 515 | 899 | 536 | 234 | 98 |
| Prostate | 636 | 684 | 188 | 157 | 106 |
| Rectum wall | 248 | 528 | 309 | 253 | 169 |
| Salivary gland | 1351 | 814 | 417 | 217 | 175 |
| Seminal vesicles | 77 | 73 | 50 | 20 | 10 |
| Skeletal muscle | 238 | 316 | 140 | 76 | 144 |
| Skin-albino | 338 | 344 | 356 | 283 | 209 |
| Small intestine wall | 827 | 1402 | 349 | 179 | 123 |
| Spinal cord | 322 | 176 | 57 | 22 | 17 |
| Spleen | 5076 | 4002 | 769 | 294 | NP |
| Stomach wall-gastric | 562 | 888 | 410 | 106 | 94 |
| Stomach wall-non-gastric | 223 | 540 | 351 | 140 | 116 |
| Testes | 323 | 923 | 343 | 154 | 96 |
| Thymus | 529 | 468 | 182 | 99 | 76 |
| Thyroid | 4844 | 7390 | 79052 | 25723 | 24053 |
| White fat | 225 | 199 | 97 | 45 | 23 |

**Suppl. Table S5B:** Concentration (ng equiv/g) of total radioactivity in selected tissues at 0.25, 1, 24, 72 and 168 h in females; C_max_ for each tissue is highlighted in yellow.

| **Tissue** | **0.25 h** | **1 h** | **24 h** | **72 h** | **168 h** |
| --- | --- | --- | --- | --- | --- |
| Adrenal-cortex | 6923 | 4345 | 785 | 485 | 159 |
| Adrenal-medulla | 8831 | 5745 | 1138 | 644 | 224 |
| Adrenal-whole | 7395 | 4710 | 928 | 514 | 186 |
| Bladder wall | 636 | 2465 | 731 | 659 | 194 |
| Blood | 13691 | 9544 | 1436 | 900 | 379 |
| Bone marrow | 3688 | 2269 | 468 | 198 | 80 |
| Brain | 176 | 124 | 24 | 19 | 6 |
| Brown fat | 1587 | 968 | 299 | 237 | 225 |
| Eye-lens | 56 | 58 | 48 | 6 | 7 |
| Eye-uveal tract | 527 | 714 | 568 | 89 | 120 |
| Eye-whole | 180 | 190 | 176 | 33 | 28 |
| Harderian gland | 632 | 666 | 357 | 179 | 53 |
| Heart muscle | 2122 | 2990 | 439 | 344 | 139 |
| Kidney-cortex | 6916 | 8186 | 595 | 350 | 147 |
| Kidney-medulla | 8092 | 8246 | 796 | 619 | 215 |
| Kidney-pyramid | 9768 | 14443 | 1056 | 644 | 143 |
| Kidney-whole | 7422 | 7771 | 674 | 428 | 153 |
| Lachrymal gland | 693 | 660 | 365 | 150 | 75 |
| Large intestine wall | 338 | 842 | 370 | 217 | 73 |
| Liver | 16578 | 9969 | 1163 | 558 | 207 |
| Lung | 8976 | 6643 | 1071 | 707 | 301 |
| Lymph node | 1103 | 1245 | 365 | 430 | 170 |
| Ovary | 6953 | 6447 | 1021 | 678 | 282 |
| Pancreas | 1296 | 986 | 287 | 172 | 64 |
| Pineal body | 2460 | 3199 | 509 | 335 | 103 |
| Pituitary | 1823 | 2728 | 405 | 272 | 109 |
| Preputial gland | 665 | 722 | 269 | 215 | 142 |
| Rectum wall | 308 | 376 | 277 | 200 | 96 |
| Salivary gland | 857 | 962 | 328 | 273 | 113 |
| Skeletal muscle | 278 | 154 | 90 | 75 | 29 |
| Skin-albino | 370 | 418 | 325 | 309 | 123 |
| Small intestine wall | 609 | 2035 | 259 | 137 | 76 |
| Spinal cord | 313 | 255 | 36 | 24 | 10 |
| Spleen | 6289 | 3263 | 606 | 290 | 104 |
| Stomach wall-non-gastric | 1141 | 1138 | 408 | 179 | 71 |
| Thymus | 669 | 629 | 138 | 147 | 55 |
| Thyroid | 3018 | 7923 | 37260 | 47883 | 18960 |
| Uterus | 852 | 836 | 212 | 358 | 93 |
| White fat | 177 | 160 | 64 | 65 | 38 |

**Suppl. Table S6:** Cumulative excretion of total radioactivity; results are expressed as % administered dose (mean of n=2).

| **Sample** | **Timepoint (h)** | **Males** | **Females** |
| --- | --- | --- | --- |
| **Urine** | 6 | 29.6 | 36.6 |
|  | 24 | 51.2 | 57.4 |
|  | 48 | 54.8 | 61.1 |
|  | 72 | 57.2 | 63.0 |
|  | 96 | 58.8 | 64.4 |
|  | 120 | 60.3 | 65.6 |
|  | 144 | 60.8 | 66.1 |
|  | 168 | 61.2 | 66.5 |
| **Feces** | 24 | 1.2 | 2.6 |
|  | 48 | 1.4 | 2.8 |
|  | 72 | 1.6 | 3.1 |
|  | 96 | 1.9 | 3.1 |
|  | 120 | 2.1 | 3.2 |
|  | 144 | 2.1 | 3.3 |
|  | 168 | 2.2 | 3.3 |
